# Preventive Medicine at Panama

**Published:** 1908-09

**Authors:** Frederick Treves

**Affiliations:** Bt., G.C.V.O.


### Preventive Medicine at Panama

By SIR FREDERICK TREVES, Bt., G.C.V.O., C.B., LL.D.

[Proceedings Royal Society of Medicine, June, 1908.]

THE Isthmus of Panama is at this moment the scene of an enterprise in sanitation of surprising magnitude, an enterprise which serves to display the forces of Preventive Medicine on a scale never before paralleled.

I visited the isthmus in February of last year and had the advantage of seeing this remarkable work under the guidance of Colonel Gorgas, the chief sanitary officer. To Colonel Gorgas is due the credit of an undertaking which in its aims and its results is not one whit less astonishing than the work of connecting by means of a canal the two great oceans of the world. Colonel Gorgas is clearing of disease one of the most pestilential spots in the tropics, and is making of the same a place where men can live in safety and in reasonable health. He is at the same time demonstrating practically the soundness and efficiency of the most recent claims of Preventive Medicine.

The isthmus is situated near to the Equator, the city of Panama standing in about the latitude of 9° N. This part of the world, ever since its discovery by Columbus, has been more or less notorious for its unhealthiness. Enriquez de Guzman, who came here in 1534, says that of every 100 men who went to Peru by way of the isthmus eighty never returned. The mortality among the Spanish gold trains was known to be very high. Equally disastrous did the isthmus prove to the hordes of men who passed westward on their way to the goldfields of California. The number of laborers who died annually during the construction of the canal by the French company is not known, but the mortality was so high that on more than one occasion the work had almost to cease owing to the ravages of yellow fever. The deaths must have amounted to many thousands. So high was the death-rate among the laborers who constructed the Trans-Isthmian Railway that it has been said—probably with some truth—that every sleeper beneath the lines represents a human life. This railway was completed in 1855. The chief causes of the great mortality on the isthmus have been yellow fever, malaria and dysentery, with occasional outbreaks of smallpox. It may be called to mind that Sir Francis Drake contracted on the isthmus the dysentery of which he died, and that he lies buried just off the coast. It was on the isthmus also that his brother succumbed to yellow fever.

The isthmus, at its narrowest part, is about thirty-three miles in a direct line. The railway, which follows a winding course, covers 47½ miles from Colon to Panama. Along the isthmus and parallel to its shores runs a ridge of hills, the ultimate offshoot of the Andes. This line of high ground is nearer to the Pacific than to the Caribbean Sea, the Culebra Pass, through which the canal has to make its way, being some ten miles from Panama. The rainfall on the Pacific side is from 50 in. to 60 in. annually; while on the Atlantic side it ranges from 100 in. to 150 in. The mean temperature of the district may be taken as 82° F., the mean humidity as 88. The tide on the Pacific coast rises 14 ft., while on the Atlantic shore the rise is only 14 in. The country for the most part is covered by dense jungle, while the lowlands, especially on the northern side, are occupied by extensive swamps. The highlands present bare and open country with extensive tracts of prairie, grass downs and breezy slopes. The denseness of the forest tracts may be illustrated by the fact that Dampier when he crossed the isthmus in 1681, with Wafer, the surgeon, and forty-four pirates, only made on an average five miles a day. He attempted a wider part of the isthmus and followed a devious course, so that the traverse of 110 miles occupied twenty-three days. Major Ronald Ross, speaking of sanitation in Panama, says: "The country is one of the worst to deal with which I have ever seen."[1]

The unhealthiness of the Panama area has been due, as has been already said, in the main to yellow fever and malaria. The actual mortality from these causes during the French occupation has never been published, but the fatality of these diseases can be to some extent gauged by the records of the British Army in the adjacent West Indies. Sir John Moore's garrison on St. Lucia amounted in June, 1796, to 4,000 men. By November the force had been reduced to 1,000 fit for duty and 1,500 sick. The campaign that lasted from 1793 to 1796 resulted, writes Fortescue, "in the total of 80,000 soldiers lost to the service, including 4,000 actually dead, the latter number exceeding the total losses of Wellington's army from death, discharges, desertions and all causes from the beginning to the end of the Peninsular War." The mortality was highest during the year 1794, when, of General Grey's original force of 7,000 men, no less than 5,000 perished during the course of the twelve months. It is probably beneath the mark, says Fortescue, that 12,000 Englishmen were buried in the West Indies during this single year. In 1780, four newly raised regiments were ordered to Jamaica. They stopped on the way at St. Lucia, where they contracted yellow fever. By the time the transports reached Kingston they had lost 168 men by death and had 780 on the sick list.

1. Lancet, 1907, ii., p. 886.

During the course of the first five months at Jamaica 1,100 more of the survivors had died of fever. It was then that Dalling, the Governor, placed the matter before the Secretary of State in the following words: "Considered only as *an article of commerce,* these 1,100 men have cost £22,000, a sum which, if laid out above ground, might have saved half their lives." In this sentence lies no little of the secret of the great success of the Americans in Panama. In addition to the dictates of humanity they have realised the part played by the laborer as an "article of commerce." The realisation of this fact by governments engaged in great enterprises of either war or peace comes, unfortunately, very late in the history of State-controlled sanitation.

The Isthmian Canal Commission was created in May, 1904. The commissioners found on the canal area a condition of chaos: the plant neglected, the district overgrown by tropical vegetation, little, if any, attempt at sanitation, and inadequate accommodation for the men employed. They found 3,000 laborers—mostly Jamaican negroes—still engaged on the works, and two French doctors, one at Panama and the other at Culebra. The Commission obtained from the Republic of Panama a grant in perpetuity of the land now known as the Canal Zone. This strip of land is ten miles wide—the line of the canal being in the center—and extends from sea to sea. Over the Canal Zone the United States have practically as complete control as if the territory were part of the home country, maintaining within its limits their own police and governing by their own laws. The grant included the group of islands in the Bay of Panama, but did not include the towns of Colon and Panama, although they are both on the canal strip. Colon, at the time of the occupation, had a population of 6,000 and Panama of 18,000. The position of these two towns, however, is defined in the following article. "The Republic of Panama agrees that the cities of Panama and Colon shall comply in perpetuity with the sanitary ordinances, whether of a preventive or curative character, prescribed by the United States, and in case the government of Panama is unable, or fails in its duty, to enforce this compliance of the cities of Panama and Colon with the sanitary ordinances of the United States, the Republic of Panama grants to the United States the right and authority to enforce the same." A like authority is granted to the United States to maintain public order in the two cities, should the Republic not be able, in the judgment of the United States, to maintain such order. The United States, moreover, obtained the power to drain these two cities, to provide them with a water supply, and to levy a water and sewage rate to defray the cost of the same.

It may be said that the sanitation of the two cities at the time of the creation of the Commission was that of the Middle Ages. Water was obtained from rain-butts and shallow wells; there was no attempt at drainage, and the disposal of refuse was left to the individual householder.

The Commission realised immediately that if the canal was to be constructed, "thorough sanitation was the first essential." In every published report sanitary measures occupy the most prominent position. The medical officer of health was allowed absolute powers; he was assured (1905 Report) that "the entire resources of the Commission" were at his disposal, and funds were immediately forthcoming for all such undertakings as he considered necessary.

The views of the Commission on this question are expressed in the following words: "The importance of completing the sanitation of the Isthmus of Panama can hardly be exaggerated, for upon it depends not only the construction of the Isthmian Canal, but also the utility of the canal when completed, and the question as to whether the canal is to be a blessing or an affliction upon the inhabitants of the earth." It was realised that unless yellow fever was stamped out the canal would become the means of carrying that disease eastwards, since the lifetime of the stegomyia has been shown to be about three months.

Sanitary works were among the very first undertaken by the Commission, and Colonel Gorgas must allow that his department has received throughout the most liberal and sympathetic support of the Government.

In 1905 the number of men employed on the canal and railway was 19,500. In the sanitation section, nearly 2,000 men were exclusively engaged. The death-rate for the year was 24.3 per thousand. The number of the constantly sick, 30 per thousand, and the deaths from yellow fever, 47. In 1906, the deaths due to yellow fever fell to 7, and since that time the disease has disappeared. The mortality for the year 1906 was 17.5 per thousand among the whites and 53 per thousand among the blacks. In 1907 the number of employees was 29,446. The constantly sick were 29 per thousand. The death-rate among the white population had dropped to 15.9 per thousand and among the blacks to 45.3 per thousand.

The sanitary works commenced in 1904, and since then developed or completed, have been upon the following lines:

In the first place the housing of the employees was taken in hand. Excellent houses, barracks, boarding-houses and hotels were built along the canal tracks. The rooms are lofty, light and well ventilated, while all are screened with copper gauze. They are provided with modern sanitary conveniences, with a good water supply and with modern plumbing. Better dwellings for a tropical climate with a heavy rainfall could hardly be designed. The feeding of the laborers has been a matter of especial care and of exceptional difficulty, on account of the fact that the bulk of the supplies have to come from the States. Numerous public kitchens and restaurants have been established where excellent food can be obtained at a minimum cost. As the West Indian negro is apt to feed himself meanly in order to save money, his wages are paid partly in board, so the security is obtained that he is amply fed. Previous to this arrangement many of the men almost starved themselves, and became thereby reduced in efficiency and in health. Ample holidays and rest days are instituted; reading rooms have been established along the zone, and clubs founded for every kind or recreation which is possible in a hot climate. A vessel is employed for free excursions to the island of Taboga, in the Bay of Panama, and every step is taken to keep the men upon whom the success of this great work depends in sound condition. One of the earliest matters undertaken was the providing of accommodation for the sick. The hospital at Ancon was greatly enlarged, and other hospitals built where required along the Canal Zone. The hospital at Ancon is a model building of its kind, replete with every modern appliance, and, indeed, as well equipped as any first-class European hospital. It is served by a specially selected staff, and in connection with the institution are ample laboratories for bacteriological and pathological work, for the chemical analysis of foods, and the like, and for general investigations in connection with the sanitation of the district. Looking back some ten years, it is scarcely to be believed that a body of engineers entrusted with the most stupendous construction of modern times should have recognised that among the *first* requirements to ensure success was a bacteriological laboratory. Colonel Gorgas, the chief sanitary officer, had further to secure proper hospital accommodation for the sick poor of the two cities, for the lepers, and for the insane. Such lepers as were unable to work lived in wretched hovels on the beach, where they existed in much the same way as the land crab. The insane poor were allowed to roam over the land or were looked after by their freinds. If they became violent they were placed in the stocks or were cast into the city prison. The Commission has now provided both lazar houses and lunatic asylums. The hospital accommodation available on the canal area amounted in 1907 to 1,845 beds.

Then came the great undertaking of making reservoirs and of providing Panama and Colon with a good and constant water supply. As soon as this work was accomplished the numerous shallow wells were filled in, water-butts, tanks and cisterns were removed, or, if left, were carefully covered in. Thus, in the year 1906, 307 wells were filled in in Panama City alone, while in the two towns 23,031 tanks or water barrels were dealt with. There followed upon this the still more extensive work of draining both the cities and carying out a modern scheme of sewage disposal, of connecting the individual houses with the sewers, of introducing water-closets, and filling in the innumerable cesspools. It is very noteworthy with what determination the Commission insisted upon the carrying out of the sanitary orders they had imposed. For example, in 1906 the canal zone police made no less than 584 arrests for violation of sanitary regulations, while in 1907, 925 persons were arrested for the same offense. In the criminal statistics for the latter year it will be observed for purposes of comparison that the charge of "disorderly conduct" heads the list with 1,176 arrests; then comes "violation of sanitary regulations" with 925; and in the third place "drunk and disorderly," with 787 arrests. Another great work undertaken by the Commission was the paving of the public ways in the two cities, and the levelling and draining of the roads. The state of the streets in Colon and Panama in 1904 was no better and no worse than that to be found in any of the old cities on the Spanish Main or in the adjacent islands. Those who would form an idea of the condition of these highways should visit Cartagena on the mainland, or the famous city of San Domingo on the island of Haiti. They would find there the surface of the main street as full of holes and gulleys as the bed of a dry torrent. To drive along these streets is an experience not to be forgotten. After a shower of rain the highway is a waste of mud interspersed by a hundred pools, which in the rainy season are never dry. The practice, moreover, of throwing all odd garbage into the streets makes their condition inconceivable. During the dry season these public ways are heavy with dust. In some of the smaller lanes about Cartagena the dust is as thick as the sand on a beach, and is only kept in check by the happy practice of emptying all slop water into the highway. To their other responsibilities the Canal Commission added the cleaning of the streets and the removal and destruction of refuse.

The most interesting work, however, undertaken by Colonel Gorgas and his staff was a crusade against the prevailing diseases on the isthmus. Of these the most important are yellow fever and malaria. Against yellow fever the inhabitants of the isthmus are immune, but they are not immune from malaria. Some 70 per cent. of the natives are the subjects of the latter disease, and it has proved most fatal. Taking the year 1907 as an example, the mortality lists on the Canal Zone present the following features: The total number of deaths was 3,822. The chief contribution to this number was made by pneumonia, which accounted for 716 deaths. The liability of the negro to pneumonia is well known, and the prominence of this disease throughout the West Indian islands is very striking. The fact that pneumonia heads the death-list in every year has no doubt suggested to the Commission that a crusade against this malady is a pressing matter. No data are forthcoming to explain the prevalence of pneumonia in the islands. The negro spends practically the whole of his day out of doors in a warm atmosphere, which is subject to but little variation of temperature the year through. At night he retires to his tiny cabin, the windows and doors of which he literally seals up; and when the number of human beings who may occupy one of these cabins during the night is noted, it is astonishing that they do not die of mere suffocation. This habit of the negro of hermetically sealing his cabin at night appears to be due solely to his fear of jumbies or ghosts, which are very troublesome on the Caribbean coast and can enter through the smallest chink. On the isthmus the houses provided for the laborer afford the amplest cubic space per man and are perfectly ventilated. They are screened with copper gauze, the meshes of which are too fine to admit even the slenderest jumbie. It is evident, therefore, that the home cabin of the negro cannot wholly explain his liability to pneumonia, since it follows him to the isthmus. The next disease in the mortality list is malaria, which in 1907 was answerable for 605 deaths. Then come the following in order: Tuberculosis of the lungs, 304 deaths; enteric fever, 150 deaths; Bright's disease, 137 deaths; diarrhea and enteritis (mostly among children), 136 deaths; dysentery, 118 deaths. In this year the number of deaths from smallpox was three. The liability of the negro to acute nephritis is well known and is shown by forty-eight deaths in 1907 and sixty-four in 1906 from this cause. To beriberi are ascribed 111 deaths in 1906 and fifty-nine in the year following. This outline of the death-rate may be completed by adding that in 1907, 236 deaths were due to accident or violence, including eight suicides.

The plan of campaign against yellow fever is as follows: The houses are in the first place screened. This screening is very complete. In the better residences not only are all the windows and doors screened, but also the verandahs. I have lived for a fortnight in a screened house and never saw a mosquito, but was bitten when out of doors. Mosquito nets are entirely dispensed with. Within the hotel at Panama I never saw a mosquito and no nets are used. The spring doors seem to be quite efficient.

In the administration building guards are stationed at these doors to see that they are not propped open and that no one loiters in the doorway. In the fire buckets in this building larvæ are now never to be found. The stegomyia do not frequent the open country, nor do they breed in swamps or large bodies of water. They are "house dwellers" and require the protection of buildings, grass, foliage, etc. A system of house to house inspection was instituted to see that no mosquito larvæ were breeding; water-butts and tanks were destroyed or carefully covered over, while puddles in yards and elsewhere were oiled. Any subject of yellow fever was immediately isolated and "placed under a mosquito-bar." In order that no case, real or suspected, should pass unnoticed eight medical men were appointed in Panama City "to act as medical inspectors and to make a daily house to house canvass of the city, reporting all suspected cases to the Health Department." The house from which any case of yellow fever has been removed is cleaned and fumigated. It is made as nearly smoke-proof as possible, all cracks and openings are sealed with paper and paste, and each room is then fumigated with sulphur or pyrethrum. In from two to four hours the house is opened and thoroughly swept out, the sweepings being taken into the street and burned. Owing to the destructive action of sulphur, pyrethrum powder is in general use on the isthmus. As in the month of June, 1905, the number of cases of yellow fever had mounted to sixty-two, the fumigation of the entire city of Panama was resolved upon. Since twelve days must elapse after the mosquito has bitten a fever patient before it can transmit the disease, it was desired to complete the work within that period. It occupied, however, forty-four days. It is impossible not to admire the docility of the people of Panama, especially as they are themselves immune, and to note that even as late as 1907 no less than fifty-nine of these citizens were fined for "having mosquito larvæ" on their premises. The average number of men employed in fumigating in Panama City alone was (in 1906) 110.

The crusade against malaria has been even more elaborate. Every new arrival on the isthmus is handed a printed circular explaining the cause of malaria and the means of its prevention, and advising the constant use of quinine in doses of at least 3 gr. a day. Quinine is placed on the table in the dining rooms and boarding camps, and large quantities of the drug are distributed broadcast. In the month of September, 1905, for example, 675,000 grs. were dispensed, mostly for prophylactic purposes. A large number of men are kept constantly employed in cutting down the dense tropical undergrowth, in mowing or burning the grass, in making and lining ditches, in filling in swamps and in oiling the surface of any pool or puddle in which mosquitoes might breed. Others are employed to inspect water tanks and barrels, to destroy such as can be dispensed with, and to screen such as are retained. As an example of the work of the anopheles brigade it may be noted that in 1906 in Colon alone the surface oiled amounted to 330,000 sq. ft. New ditches were cut to the extent of 200,000 lineal feet. Of these ditches 20,000 ft. were stoned or cemented. Two million lineal feet of old ditches were cleared, graded, stoned or filled in. The area of brush and grass cleared amounted to 21,000,000 sq. yds. Never has a crusade been carried out with such completeness, for never has a chief sanitary officer had so free a hand. It is needless to point out that the mere oiling of pools does not constitute the sole prophylactic measure against malaria. In a well-to-do town in the tropics it may be supposed that the land has been thoroughly drained and every suspected water area oiled, but there are still many varieties of vegetation which afford a breeding place for mosquitoes; as instances may be cited pines and such a palm as the traveler's palm. We may be sure that the pine grower will not sacrifice his harvest in the public interest, nor will the wealthy resident allow the palms, which are the glory of his garden, to be cut down. It is much to be hoped that a list will be forthcoming of garden and other plants in which mosquitoes breed. On the Canal Zone no such list was needed. The place denounced was swept bare.

On one point of interest the reports of the Commission are silent. They do state upon what grounds the crusade against the land crab is based. It will be noticed in the last report that in the course of the year in Cristobal alone no less than 30,566 crab holes were oiled and 10,571 crabs were killed. I am not aware that the land crab has ever been seriously studied from a medical or sanitary point of view. That the animal is a remarkable and agile scavenger is allowed; that his habits are disgusting and his place of hiding unhygienic are more or less evident; but I have not met with any account which accuses this creature of the dissemination of disease. The matter is of some interest. On the Island of Barbados, for example, are to be seen more land crabs to the square yard than I have noticed in any other part of the world, yet Barbados is a remarkably healthy island, entirely free from yellow fever and but slightly troubled with malaria. The land crab has there no price upon his head, and, except for the damage he does to gardens, graveyards, and roadsides, is not anathema.

Time will not permit of any account of the quarantine arrangements on the Canal Zone, nor of the very vigorous and successful manner in which an outbreak of bubonic plague was dealt with in 1905.

It will be seen, I hope, from the above brief description that the Isthmus of Panama provides at this moment an object-lesson which those who control the destines of men might study with advantage. It provides for the realisation of a long contemplated and heroic ideal—the medical officer of health with a free hand.

DISCUSSION.

The PRESIDENT (Dr. Newsholme) said Sir Frederick Treves had had a wonderful story to tell the Section, and those who had heard Sir Frederick before and had read his writings, would know that the story had lost nothing in the telling. The members, he was sure, were all much obliged to him. Panama was evidently an administrative Elysium for medical officers of health. The immense progress in sanitation there had been due to necessity, though that did not detract from the credit accruing for the work, and in that respect there was a parallel in our country, where cholera was the immediate determining cause of sanitary reform.

Sir SHIRLEY MURPHY said he had very much pleasure in proposing a vote of thanks to Sir Frederick Treves for his address. The subject was of entrancing interest to the section, and its attractiveness was much heightened by the manner in which it had been presented. He knew the author had experienced some difficulties in connection with preparation, but no obstacles were apparent in the reading, and the section was very grateful for the way Sir Frederick had presented the subject.

Dr. SANDWITH seconded the resolution of thanks. No one. he was sure, had enjoyed the paper more than he had, and he thought the author was a living illustration of how a man might cease to be a practitioner, might even, if he chose, cease to be a surgeon, but could not cease to be a citizen of the Empire; he might still do good work, and he (Dr. Sandwith) was glad to see that Sir Frederick had not ceased to be a teacher. The reader had made clear the different treatment meted out to the American medical officer compared with that accorded to his British confrère. At the time when yellow fever was stamped out in Havana the American medical officers were given military power, and he (Dr. Sandwith) suggested that medical officers of health in this country should try to get the same powers. It was a great surprise to him, after being some years abroad, to find the inferior position occupied by medical officers of health in this country compared with what their colleagues enjoyed abroad, for in the fight against epidemic disease it was essential to have officers who could be trusted to act. Sir Frederick Treves had spoken about yellow fever, malaria, and dysentery, and he (Dr. Sandwith) had been thinking how little the present generation realised the evils of malaria. Less than ten years ago the Egyptian army was reoccupying the Sudan, and black troops were sent up the Blue Nile because they were supposed to be less susceptible to malaria than the Egyptians. Of course they were commanded by British officers. On coming to Karkog, a village about 300 miles from Khartum, the force consisted of 451 healthy men. Seventeen days later sixty-nine of them were struck down, and in another two days 380 men, or about 84 per cent., were down with malaria, ten deaths occurring within a few days. Of the thirteen British officers one died, six were seriously ill, and six were only slightly ill. From experience in the United States, Egypt and South Africa he could confirm Sir Frederick's statement as to the liability of the black man to get pneumonia. He had not an explanation to give for it but thought there were obvious contributing factors. It was not at all unusual for a European to live for years with arrested or cured tuberculosis but the African seemed always to succumb rapidly to it and when he had pneumonia he often died of it. Again a negro suffering from typhus was more apt than a European to die of hypostatic congestion of the lungs. One reason he believed was that the black did not take full breaths; he was generally a man of the plains unaccustomed to deep breathing and seemed to have no reserve of lung power which would tide him over a respiratory illness. Also the black seemed to have very feeble resistance to tubercle bacilli and pneumococci.

The resolution was cordially carried.

Mr. Malcom Watson said they had heard that night from Sir Frederick Treves an interesting account of the success with which the Americans had dealt with malaria and yellow fever in Panama. By the request of the secretary he was there to ask them to turn to the other side of the lantern and hear how a British Government had dealt with a somewhat similar problem. Panama was about 90° W. longitude the Federated Malay States were some 90° E. They were within 5° of the Equator had a heavy rainfall amounting to about 100 in. per annum and had been notorious for a malarious climate. Early in 1901 on assuming duty as district surgeon in the district of Klang he found the hospital full of malaria; Government officers were continually off duty with the disease. The town was smothered in jungle; acres of swamp abounding with anopheles stretched along the small hills on and around which the town was built. Setting himself to collect statistics of the disease, he was soon in a position to present a report to the Government of such a nature that money for drainage works was promised for the following year. Before, however, the year was out, the town was almost devastated by the disease, and the Chinese suspended business for three days in order that all their attention might be concentrated on processions, theatres, and such other rites as were essential to the driving of the devil from the town. As a result of the drainage, malaria had almost completely disappeared from the town, only some eight or ten cases per annum being found in which it was impossible to trace infection from outside. The result of the work at Klang would have been very striking had it been alone, but at the same time as the drainage of Klang was undertaken the drainage of Port Swettenham was also carried out, with equally satisfactory results. There had thus been two entirely independent experiments. Unlike Klang, in which there were small hills, Port Swettenham was originally a mangrove swamp covered by all spring tides. It was always malarious, and early in 1901, he drew the attention of the Government to the necessity for antimalarial measures. The port was opened on September 15, 1901, and, in order to obtain statistics of the malaria, he deputed a subordinate to visit each house daily to record and treat each case of malaria as it arose. The epidemic, however, became of so severe a nature that the business of the port was completely disorganised and the propriety of closing it was discussed. A commission, consisting of Dr. H. Wright, Dr. E. A. O. Travers, the speaker and three inquirers, was deputed by Government to report on and carry out the necessary sanitary measures, and the commission carried out the measures which he had recommended some months before, viz., clearing jungle, drainage, and the like. The result of these measures was that within a few weeks the port was working smoothly and it was not found necessary to use wire gauze on the houses. From 1902 to 1907 Port Swettenham was practically free from malaria, but in 1907, as the result of the blocking of certain drains by new engineering works, a small outbreak occurred again. It was a very striking reversal of the experiment, but for the sake of the public health the drains had to be reopened as soon as the damage they were doing was shown. He did not propose to go into the statistics of the malaria of the two towns in detail, but some idea of the value of the antimalarial measures would be gathered from the following: in 1901, 610 cases of malaria were admitted to Klang Hospital; in 1902, 1903, 1904, 1905, the numbers were respectively 199, 69, 32, 23; while from the surrounding undrained area the numbers increased. Not only did the admissions to hospital for malaria diminish, but there was an extraordinary decrease in the number of deaths, not only from malaria but from all other causes registered in the town, showing to what an extent malaria predisposed persons to other diseases. The number of deaths from all causes registered in the two towns were, in 1900 and 1901, 474 and 582 respectively, and in 1902, 1903, 1904, 1905, were 144, 115, 122, 113 respectively, and again the deaths registered without the towns showed an increase. The children, too within the towns enjoyed an immunity from the disease in striking contrast to those outside; during 1904 and 1905 examinations of the children revealed no infection of those permanently resident within the towns, while in 1904, 33.89 per cent. were infected of 298 children residing outside of the towns. In 1901, Government officers received 1,026 days sick leave on account of malaria; in 1905 only thirty days leave was given, and this was given to officers who although resident in Klang, had contracted the disease outside. Finally, he would like to say a word on the measures undertaken. In 1900 there was considerable discussion as to the relative merits of mosquito-netting, quinine and mosquito destruction. He followed the lines laid down by Ross, viz., mosquito destruction, and in order to obtain permanent results adopted drainage as the method. It appeared to him to be the method for towns, because, first, it was permanent and, secondly, it could be carried out independently of the coöperation of the population.

Professor Ronald Ross, C.B., F.R.S., said that a very pleasant evening had been spent in listening to Sir Frederick Treves's most interesting description and Dr. Malcolm Watson's account of his good work, which was perhaps scarcely second in importance and elegance—if such a term were permissible in that connection—to that of Colonel Gorgas, and only in the extent to which it was carried out. The colonel had all the resources of the American Republic behind him. Dr. Watson's work had been most excellently done. The point as to the great prevalence and fatality of pneumonia had been dealt with by Dr. Sandwith. He quite agreed that that disease was frequently the terminal complaint following malaria, dysentery, and other things. The same kind of pneumonia statistics were obtained in India among the troops there, both native and European. With regard to mosquitoes in trees, he had just come from Mauritius, and it was the same there. On the average, one hole was found in every three trees; but they did not breed anophelines there, only stegomyia and culex. It was not necessary to cut down the whole tree; the simplest way was to fill up the hole with mud. His moustiquiers made a kind of concrete out of red earth and lime, which was placed in the hole, and he believed it effectually stopped it for a long time. One of the mosquito destroyers had to make a tour of the trees once a year and stop them up; but those holes did not cause much sickness in Mauritius, because they did not breed the anophelines. The same remark applied to land crabs and their holes. In Mauritius it was found to be only the big marsh which caused malaria, not a few pots full of water. It was true that the anophelines bred in small collections of water, but for an outbreak it was necessary that there should be many such small collections of water. In Mauritius a careful observation was kept of the rate of fall in the disease at different distances from a marsh. A few yards from the marsh the spleen involvement rate was 95 per cent.; a few hundred yards away the rate fell quite rapidly, and further away it was as low as 30 per cent. or less of the children. The question of circumvallation was a very important one. With regard to the use of petroleum, he did not think it was worth while spending much on that for malaria; drainage was best. Labor could be best employed in clearing waters and draining. He agreed with Dr. Watson's statement that engineers were sometimes very troublesome in connection with operations such as had been described. There were many other points, all of great interest to him, on which he could talk for a long time, but the hour was late, and he must forbear.

Sir FREDERICK TREVES, in reply, said. With regard to the tree question, he asked Major Ronald Ross whether such a tree as the Madagascar or traveler's palm was not a serious breeding ground for the anophelines and other mosquitoes. When he, Sir Frederick, was in Trinidad there was an outbreak of yellow fever there, and that tree was thought by some to play a possible part in it. In their radical measures the Americans realised that there were other collections of water besides those on the face of the ground in which mosquitos might breed. Colon presented the additional difficulty that the swamp in some places was below the sea level. The Americans had another advantage in their sanitary work which was exceptional, *i. e.,* the privilege of what was called "dumping." The enormous mass of earth taken from the cutting could be dropped into the swamp. Thus the swamp at the back of Colon, which could hardly be drained, was being closed by being made a dumping place.

Colonel MACPHERSON, in continuing the discussion, gave some interesting and rather new facts about the subject of the paper, and said his excuse for doing so must be that he returned from Panama only last month, after studying, with Colonel Gorgas, the subject of Sir Frederick Treves's lecture. The reader mentioned that the Commission on Sanitation had absolute powers from the commencement of the work, but that was not so. Colonel Gorgas came from Havana, where he had extinguished yellow fever, in the year 1901, in eight months. He came to Panama, where to his astonishment, he found it took two years to blot out the disease. He told Colonel Macpherson that the probable reason for that difference was that when he started the campaign in Havana he had the advantage of two years of previous sound sanitary work and organisation in the city, whereas in Panama he had to start at the beginning, and had had very great difficulty in obtaining necessary supplies and authority to carry out thorough sanitary measures, even from the people who ought to have supported him. The chief engineer, the Governor, and the chairman of the Canal Commission had all joined in a recommendation that Colonel Gorgas's work should be discontinued—they said it was not practicable, or at any rate could not be put into practical execution. The object-lesson pointed by the facts was what Colonel Gorgas had set himself to do was practicable, and that it was the result of determining to triumph over obstacles. He was sure that a remark contained in a letter of Colonel Gorgas's to him—namely, that "successes on these lines would make the work of future sanitarians easier," was one which all would agree with. He (the speaker) had carefully studied the cost of such work, and it was not so great as would be imagined. Colonel Gorgas received $2,000,000 for extinguishing the disease in that zone, and that included all the hospital work for the treatment of the sick, as well as measures for the prevention of disease. The amount spent in actual prevention was only $500,000 per annum. That worked out at an insurance of 2½ per cent. on the estimated cost of the canal for preventive work alone. Another interesting point about the sanitation at Panama was that it was despotic hygiene. President Roosevelt had told him that he could not get the same results in the best cities of the States as he could in Panama, simply because of that system of despotic hygiene. What Sir Frederick said about the punishments meted out to those who had larvæ in their houses seemed surprising, but it was perfectly true. In Colon, occupiers of houses were fined 50s. if larvæ were found in the houses, but the same man had never to be fined twice. In regard to pneumonia, Colonel Gorgas had appointed a commission of medical officers and inspectors of health to go into that, and they studied the subject very thoroughly. Only one fact seemed to be established—namely, that negroes who got pneumonia always did so within three months of coming to the Canal Zone, and this pneumonia seemed to be the general sequel to attacks of a catarrh of the respiratory passages of the nature of influenza. The draining of the swamps, so as to get rid of malaria, was the chief trouble in the zone at present. Colonel Gorgas adopted the means shown in Dr. Watson's photographs—namely, draining along the contours—not along the center—and in oredr to minimize the cost of keeping drains clear he had begun to lay agricultural drains along the line of drainage trenches, covering them with loose stones and clinkers. The anopheles would not grow unless there was vegetation along the banks of the streams, drains, or pools, and no vegetation grew where this method was adopted.

